# Supplementary material for: The in vivo measurement of replication fork velocity and pausing by lag-time analysis
Source: Nat Commun. 2023 Mar 30;14:1762. doi: 10.1038/s41467-023-37456-2 (PMC10063678; doi:10.1038/s41467-023-37456-2)
Supplement: Supplementary file 2 — Description of Additional Supplementary Files [file 41467_2023_37456_MOESM2_ESM.pdf]

Description of Additional Supplementary Files

File Name: Supplementary Data 1
Description: A comprehensive table of measured locus-dependent fork velocities and pause durations for datasets analyzed in the paper.

File Name: Supplementary Movie 1
Description: Simulated stochastic marker frequency evolution for the Terminus 1 Model. The marker frequency (copy number) is shown as a function of genomic position. The movie's time is shown in simulation units. The dynamics emphasizes the importance of performing the calculations of the marker frequency in the steady-state limit.

File Name: Supplementary Movie 2
Description: Simulated stochastic marker frequency evolution for the Terminus 3 Model. The marker frequency (copy number) is shown as a function of genomic position. The movie's time is shown in simulation units. The dynamics emphasizes the importance of performing the calculations of the marker frequency in the steady-state limit.
