# Supplementary material for: The in vivo measurement of replication fork velocity and pausing by lag-time analysis
Source: Nat Commun. 2023 Mar 30;14:1762. doi: 10.1038/s41467-023-37456-2 (PMC10063678; doi:10.1038/s41467-023-37456-2)
Supplement: Supplementary file 6 — Reporting Summary [file 41467_2023_37456_MOESM6_ESM.pdf]

nature portfolio

Corresponding author(s): Paul A. Wiggins

Last updated by author(s): Feb. 16, 2023

Reporting Summary

Nature Portfolio wishes to improve the reproducibility of the work that we publish. This form provides structure for consistency and transparency in reporting. For further information on Nature Portfolio policies, see our Editorial Policies and the Editorial Policy Checklist.

Statistics

For all statistical analyses, confirm that the following items are present in the figure legend, table legend, main text, or Methods section.

n/a	Confirmed	
☐	☒	The exact sample size (n) for each experimental group/condition, given as a discrete number and unit of measurement
☒	☐	A statement on whether measurements were taken from distinct samples or whether the same sample was measured repeatedly
☐	☒	The statistical test(s) used AND whether they are one- or two-sided<br>*Only common tests should be described solely by name; describe more complex techniques in the Methods section.*
☒	☐	A description of all covariates tested
☒	☐	A description of any assumptions or corrections, such as tests of normality and adjustment for multiple comparisons
☐	☒	A full description of the statistical parameters including central tendency (e.g. means) or other basic estimates (e.g. regression coefficient) AND variation (e.g. standard deviation) or associated estimates of uncertainty (e.g. confidence intervals)
☐	☒	For null hypothesis testing, the test statistic (e.g. F, t, r) with confidence intervals, effect sizes, degrees of freedom and P value noted<br>*Give P values as exact values whenever suitable.*
☒	☐	For Bayesian analysis, information on the choice of priors and Markov chain Monte Carlo settings
☒	☐	For hierarchical and complex designs, identification of the appropriate level for tests and full reporting of outcomes
☒	☐	Estimates of effect sizes (e.g. Cohen's d, Pearson's r), indicating how they were calculated

Our web collection on statistics for biologists contains articles on many of the points above.

Software and code

Policy information about availability of computer code

Data collection	Simulated cell growth data were generated in MATLAB R2022b using the Gillespie algorithm (Gillespie 1977).
Data analysis	All data analysis scripts were written in MATLAB R2022b. Sequencing reads were mapped using the Bowtie 2 (v2.4.5) Support Package from the MATLAB Bioinformatics Toolbox (v4.16.1). SAM files were processed using the command-line tool SAMtools (v1.16.1). Fit parameters were optimized using the MATLAB Optimization Toolbox (v9.4).

For manuscripts utilizing custom algorithms or software that are central to the research but not yet described in published literature, software must be made available to editors and reviewers. We strongly encourage code deposition in a community repository (e.g. GitHub). See the Nature Portfolio guidelines for submitting code & software for further information.

Data

Policy information about availability of data

All manuscripts must include a data availability statement. This statement should provide the following information, where applicable:

- Accession codes, unique identifiers, or web links for publicly available datasets
- A description of any restrictions on data availability
- For clinical datasets or third party data, please ensure that the statement adheres to our policy

The sequencing datasets generated during the current study are available from the NCBI Sequence Read Archive with the BioProject accession code PRJNA919081. The data from Galli et al. [35] and Midgley-Smith et al. [4] are both available from the European Nucleotide Archive (ENA), with the accession codes PRJEB28538 and

PRJEB25595, respectively. The digitized data from Wang et al. [32] and Srivatsan et al. [31] are available in the Source Data file. More detailed information about data availability is provided in Supplementary Table 2.

Human research participants

Policy information about studies involving human research participants and Sex and Gender in Research.

Reporting on sex and gender	We have not conducted any research on human participants.
Population characteristics	We have not conducted any research on human participants.
Recruitment	We have not conducted any research on human participants.
Ethics oversight	We have not conducted any research on human participants.

Note that full information on the approval of the study protocol must also be provided in the manuscript.

Field-specific reporting

Please select the one below that is the best fit for your research. If you are not sure, read the appropriate sections before making your selection.

☒ Life sciences ☐ Behavioural & social sciences ☐ Ecological, evolutionary & environmental sciences

For a reference copy of the document with all sections, see nature.com/documents/nr-reporting-summary-flat.pdf

Life sciences study design

All studies must disclose on these points even when the disclosure is negative.

Sample size	The total number of reads collected per genome for this study was roughly 6 x 10^7. The number of reads per genome for the other sequencing datasets used was roughly 2 x 10^7. These sample sizes represent the number of sequence reads that were mappable to the reference genome for each deep sequencing experiment. Our analysis includes a statistical model selection step, where the Akaike Information Criterion was used to determine the optimal number of parameters needed to fit the data. This selection step takes into account the signal and noise levels of each individual dataset, so our analysis adapts directly to the sample size. Hence, the sample size is sufficient for the analysis.
Data exclusions	No data were excluded; however, regions of the genome that could not be uniquely mapped to the reference genome are not analyzed.
Replication	For next-generation-sequencing and growth rate data collected for this study, three replicates were collected. All attempts at replication were successful.
Randomization	Our research did not involve treatment groups and therefore no random assignments could be made.
Blinding	Blinding was not applicable to our study. There were no "participants" and confirmation bias was avoided by testing hypotheses, motivated by data, on new experiments.

Reporting for specific materials, systems and methods

We require information from authors about some types of materials, experimental systems and methods used in many studies. Here, indicate whether each material, system or method listed is relevant to your study. If you are not sure if a list item applies to your research, read the appropriate section before selecting a response.

Materials & experimental systems

n/a	Involved in the study	
☒	☐	Antibodies
☒	☐	Eukaryotic cell lines
☒	☐	Palaeontology and archaeology
☒	☐	Animals and other organisms
☒	☐	Clinical data
☒	☐	Dual use research of concern

Methods

n/a	Involved in the study	
☒	☐	ChIP-seq
☒	☐	Flow cytometry
☒	☐	MRI-based neuroimaging
